# Machine Learning-Enabled Optimization and Prediction of Mechanical Properties of 3D-Printed PLA Composites Filled with Rice Husk Biochar

**DOI:** 10.3390/polym18040527

**Published:** 2026-02-21

**Authors:** Borhen Louhichi, Joy Djuansjah, P. S. Rama Sreekanth, Sundarasetty Harishbabu, P. V. Subhanjaneyulu, Santosh Kumar Sahu, It Ee Lee, Gwo Chin Chung

**Affiliations:** 1 Engineering Sciences Research Center (ESRC), Deanship of Scientific Research, Imam Mohammad Ibn Saud Islamic University (IMSIU), Riyadh 11432, Saudi Arabia; 2 College of Engineering, Imam Mohammad Ibn Saud Islamic University (IMSIU), Riyadh 11432, Saudi Arabia; 3 School of Mechanical Engineering, VIT-AP University, Besides A.P. Secretariat, Amaravati 522237, Andhra Pradesh, India; 4 Faculty of Artificial Intelligence and Engineering, Multimedia University, Cyberjaya 63100, Malaysia; 5 Centre for Smart Systems and Automation, COE for Robotics and Sensing Technologies, Multimedia University, Cyberjaya 63100, Malaysia; 6 Centre for Wireless Technology, COE for Intelligent Network, Multimedia University, Cyberjaya 63100, Malaysia

**Keywords:** 3D printing, rice husk biochar (RHBC), CCD, ANOVA, machine learning

## Abstract

This investigation focuses on rice husk biochar (RHBC) as a sustainable filler in a polylactic acid (PLA) matrix. This study employs optimization techniques, including central composite design (CCD) and analysis of variance (ANOVA), to systematically evaluate the effects of key 3D printing parameters such as filler content (0 wt.%, 10 wt.%, 20 wt.%), nozzle temperature (190 °C, 200 °C, 210 °C), orientation angle (0°, 60°, 120°), and fill pattern (hexagon, triangle, and 3D infill). Furthermore, machine learning models are used to predict the mechanical properties of PLA/RHBC composites from experimental data. The effects of these parameters on tensile strength, Young’s modulus, and hardness were analyzed. The ANOVA results showed that filler content was the most influential factor for tensile strength and Young’s modulus, contributing 36.47% and 73.25%, respectively, compared to pure PLA. For hardness, both filler content and nozzle temperature were key contributors, with a 44.08% improvement over pure PLA. Machine learning models, including multiple linear regression (MLR), K-Nearest Neighbors (KNN), Support Vector Machine (SVM), and Gradient Boosting, were used to predict the mechanical properties. Among these, Gradient Boosting achieved the best performance, with R^2^ values of 97.79% for tensile strength, 98.79% for Young’s modulus, and 96.8% for hardness. This study provides a robust framework that combines experimental analysis, statistical design, and machine learning to optimize RHBC as an eco-friendly filler for the development of PLA composites for adoption in the automotive, sports and aerospace industries.

## 1. Introduction

Thermoplastic polymers are an integral part of modern society due to their versatility, affordability, and durability [1,2]. However, the use of thermoplastic polymers has led to serious environmental consequences, underscoring the need for alternative solutions [3]. Polylactic acid (PLA) is a biodegradable thermoplastic polymer derived from renewable resources such as corn starch and sugarcane and presents a promising solution to the above-mentioned issue [4,5]. At the same time, PLA has its own limitations due to its low tensile and hardness properties, which hinder its wide range of applications. To overcome these challenges, reinforcing PLA with biofillers offers a viable solution. Among various biofillers, biochar stands out as an ideal choice due to its low cost and easy availability. Biochar, a carbon-rich material derived from organic waste, can enhance PLA’s durability while boosting its eco-friendly benefits, making it a more sustainable alternative to conventional plastics [6]. It was established that the mechanical properties of coconut biochar 10 wt.% filler in polylactic acid (PLA) matrix enhanced the tensile strength by 223% and the hardness by 89% compared to pure PLA [7]. Tomato stem waste used to produce biochar as a filler in PLA at 5 wt.% and 7.5 wt.% enhanced the flexural strength and yielded a reduction in tensile strength at 5 wt.% [8]. In another study, it was noted that there is an enhancement of tensile strength and durability in composites made from polypropylene (PP), polycaprolactone (PCL), and PLA filled with dairy manure or wood chips [9]. The addition of rice husk biochar at 1, 2, 3, and 5 wt.% to PLA using the L16 orthogonal array resulted in an enhancement in tensile strength to 36 MPa and Young’s modulus to 1103 MPa [10]. The incorporation of carbon-rich biochars into PLA at 0.25 wt.% enhanced mechanical properties, particularly impact strength and the modulus of elasticity [11]. A beech wood-derived biochar at 5 wt.%, 10 wt.%, and 20 wt.% on PLA yielded a significant enhancement of tensile strength and modulus that occurred at 5 wt.% biochar, but a higher biochar content, such as 10 wt.% and 20 wt.%, reduced mechanical properties due to poor dispersion and agglomeration [12]. The incorporation of a submicron- and micron-sized Pinus sylvestris char (PS)-filled PLA composite resulted in an enhancement of 98% in tensile strength and 25% in bending strength compared to pure PLA [13]. A betel nut shell-derived carbon (BNAC)-infused PLA composite, varying from 0025, 0.05, and 0.1 wt.% through a 3D printing route, showed an enhancement in tensile strength of 51.1% at 0.1 wt.% [14]. PLA reinforced with 20% talc particles and PLA with 5% MMT showed a positive effect on the stiffness and strength [15]. Polylactic acid (PLA) composites were prepared by incorporating rice straw hydrochar (HC) at loadings of 5, 10, 15, and 20 wt.% [16]. The addition of HC significantly enhanced the tensile modulus, which increased by 135% at a 20 wt.% HC loading compared to neat PLA. A groundnut shell-derived biocarbon (GNSC) as a reinforcement for polylactic acid (PLA) filaments was successfully fabricated through fused deposition modeling (FDM) [17]. It was observed that GNSC synthesized at 800 °C (GNSC800) showed a high carbon content and nanosized spherical particles. PLA-GNSC composite filaments with 0.25%, 0.5%, and 0.75% GNSC loading were produced, and the results showed that 0.5% GNSC significantly enhanced mechanical properties, with a 39.8% increase in tensile strength and a 17.5% improvement in tensile modulus. PLA reinforced with bamboo charcoal (BC) to form a PLA/BC composite at 2.5% to 10% weight resulted in a 43% improvement in tensile strength, 99% in flexural strength, and 52% in ductility index, with optimal results at 7.5% BC [18]. A study demonstrated that biochar, added at 1%, 2.5%, and 5% by weight, significantly enhances the mechanical properties of recycled PLA (r-PLA) composites [19]. At 2.5% and 5% biochar, it was observed that biochar increased the elastic modulus by 20%. A grapevine biochar (GVC) at 1 wt.% and 10 wt.% with particle sizes of 200 and 100 mesh was successfully synthesized [20]. It was noted that the sample’s tensile strength was 79.79 MPa at a 200 mesh size. Groundnut shell powder (GNSC) as a filler in PLA composites at 0.25%, 0.5%, and 0.75% by weight was successfully fabricated and the results confirmed that, at 0.5% GNSC, the tensile strength improved to 58.61 MPa [21].

This literature review showed that various bio-based materials and biochar, such as coconut shells, wood, bamboo, tomato stems, and rice husk, have been widely explored as fillers. Among these, rice husk biochar stands out as an ideal choice due to its low cost, high availability, and carbon-rich composition. It not only improves the mechanical properties of PLA but also promotes sustainability by utilizing an agricultural waste by-product. PLA composite fabrication via 3D printing is an advanced method. Despite its potential, the optimization of rice husk biochar-reinforced PLA composites to enhance mechanical properties via 3D printing has not been explored. Furthermore, the use of advanced statistical techniques and machine learning methods to predict these properties remains under-researched. This study aims to bridge the gap by focusing on both the optimization and prediction of rice husk biochar performance in 3D-printed PLA composites. Hence, this research offers a more comprehensive approach than traditional methods, which typically rely solely on experimental techniques or individual approaches. Ultimately, this study contributes to the development of more efficient and reliable manufacturing processes for rice husk biochar-reinforced PLA composites, thereby enhancing mechanical performance and sustainability.

## 2. Materials and Methods

### 2.1. Materials

**Figure 1:**
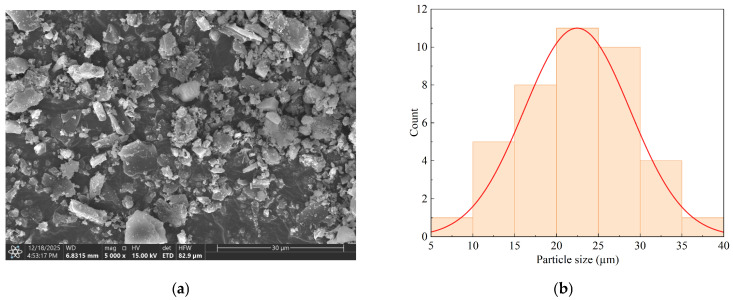
(**a**) SEM morphology of RHBC; (**b**) particle size distribution of RHBC.

Polylactic acid (PLA) granules, with a specific gravity ranging from 1.30 to 1.35 g/cc, a melt flow rate of 2–4 g/10 cc, and a tensile strength of 25–35 MPa, were supplied by Deltora Biopolymer Pvt. Ltd., Ahmedabad, India. Rice husk biochar (RHBC), characterized by fragmented and irregular shapes with a particle size of less than 40 µm, was purchased from Vistarah Innovation Pvt. Ltd., Amaravathi, India. The biochar was chemically modified with sodium hydroxide (NaOH) for surface functionalization by the supplier. Scanning Electron Microscope (SEM) (Helios Scios 2 DualBeam, ThermoFisher Scientific, Waltham, MA, USA) was used to study the morphology of the as-received RHBC, as shown in Figure 1a. The morphology of RHBC appears to have an irregular structure. Figure 1b shows the particle size distribution of RHBC which was analyzed through ImageJ software (v1.54, National Institute of Health, Bethesda, Rockville, MD, USA). This size range is significant because it can substantially affect the mechanical properties of PLA composites, including tensile strength, stiffness, and flexibility [22].

### 2.2. Statistical Analysis

#### 2.2.1. Experimental Design 

The experimental design plays a crucial role in the statistical analysis for the systematic investigation of the relationship between multiple factors, such as filler content wt.%, sample orientation angle, pattern of filling, and nozzle temperature, each at two levels as shown in Table 1, with the responses including tensile strength, Young’s modulus, and hardness of RHBC/PLA composites. In this study, the experimental design was carried out using Design Expert-13 software based on central composite design (CCD) in response surface methodology (RSM). To identify the effect of the factors, both linear and nonlinear, the experimental design with 24 randomized runs was structured [23,24].

#### 2.2.2. Analysis of Variance (ANOVA)

Analysis of variance (ANOVA) is a statistical technique used to assess the significance of factors and their interactions. In this current study, the ANOVA was performed to analyze the relation between printing factors and the corresponding responses. The quartic model is a higher-order polynomial model used for comprehensive analysis, including complex integrals such as quadratic and quartic (fourth-order) terms and linear interactions. A 95% confidence interval was used to assess the model’s significance. The model is significant or insignificant, identified based on this confidence interval [25,26].

### 2.3. Sample Preparation 

The sample preparation process is carried out by fused deposition modeling on a 3D printer by varying the printing parameters as defined in Table 1. The sample preparation involves three basic steps, which are composite preparation, filament extrusion, and sample printing.

#### 2.3.1. Composite Preparation and Filament Extrusion 

**Figure 2:**
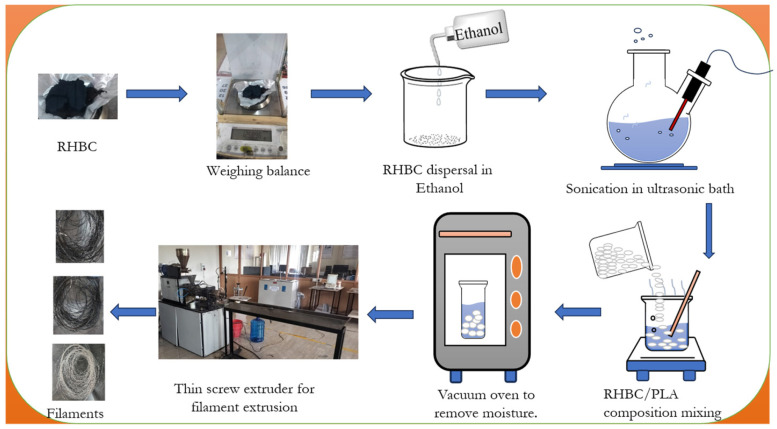
Steps involved in composition preparation and filament extrusion.

Filament extrusion is the process of converting the granular PLA and its RHBC composites to wire, as shown in Figure 2. This process involves composition preparation and filament extrusion. In the composite preparation, the PLA was reinforced with 10 wt.% and 20 wt.% alkaline-treated rice husk biochar (RHBC). The alkaline treatment removes surface impurities and increases surface roughness and active functional groups, thereby improving interfacial adhesion [27,28]. The required weight of RHBC is collected using a digital weighing balance, then mixed with ethanol at a 20:1 ratio to improve the wettability of the biochar. The mixture is sonicated for 30 min at a frequency of 40 kHz and a power of 200 W to achieve uniform dispersion of the biochar. The solution is heated with a hot plate to complete the evaporation of ethanol with continuous stirring while adding the PLA granules. The mixture was dried with a vacuum oven at 70 °C for a period of 24 h to remove the moisture. Finally, the moisture removed the RHBC/PLA composite granules used to extrude the filament with a twin screw extruder (AASAVI/25TS/CO/300/30, Aasabi Machinery Pvt. Ltd., Mumbai, India). This was operated with the barrel temperature ranging from 170 to 210 °C with a 2 mm extruder die diameter at a speed of 30 rpm to extrude the filament diameter of 1.75 ± 0.05 mm by rapidly cooling the molten composite by passing it through the cold water bath. The obtained filament was collected and stored in an airtight container to avoid moisture absorption [29,30].

#### 2.3.2. Sample Printing

**Figure 3:**
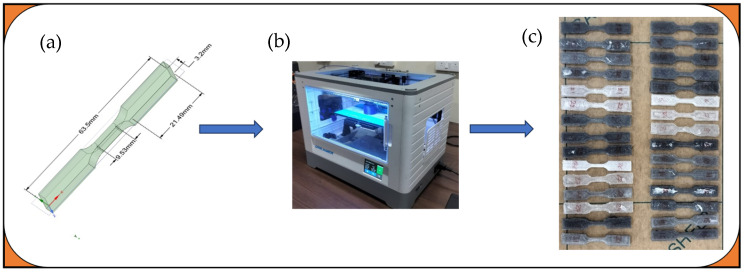
(**a**) Designed samples, (**b**) 3D printer, (**c**) printed samples.

The FlashForge FDM 3D printer with a 0.4 mm nozzle was used for printing the samples, varying the factors to control aspects such as sample orientation angle, fill pattern, and nozzle temperature as per the experimental design, outlined in Table 2. The sample geometries were designed according to ASTM D638 Type V standards for tensile testing, as shown in Figure 3a. The designed models were imported into the FlashPrint slicing software for slicing, with the printing parameters specified in Table 2. The following printing settings were kept constant throughout the experiment: layer height of 0.2 mm, print speed of 40 mm/s, bed temperature of 60 °C, cooling fan settings at 100% after the first few layers, raster width of 0.4 mm, and drying/conditioning of the filament at 50 °C for 4 h prior to printing to remove moisture. A wall thickness of 1.6 mm and 100% infill density were used to ensure solid internal structures. The printing process was carried out with the FlashForge Dreamer 3D printer (Reddx Technologies Pvt Ltd., Chennai, India), shown in Figure 3b. After printing, the samples were inspected for defects and measured for dimensional accuracy according to ASTM standards before mechanical testing, as shown in Figure 3c.

### 2.4. Experimental Analysis 

#### 2.4.1. Tensile Testing 

Tensile testing was performed to evaluate the ultimate strength and Young’s modulus of the PLA- and RHBC-reinforced composites. A universal testing machine (UTM) (H10KL, Tinius Olsen India Pvt. Ltd., Noida, Uttar Pradesh, India) was used to apply uniaxial loading to the samples, which were printed in a dog-bone shape per ASTM D638. The experiments were conducted at a crosshead displacement of 2 mm/min at room temperature. Five repetitions were performed, and the average of these repetitions was noted. The failure samples were examined using scanning electron microscopy (ZEISS Model: EVO 10, Oberkochen, Germany) to analyze the surface structure and failure mechanism. Before capturing the mechanism, the samples were coated with a thin layer of gold to prevent electrical conductivity [31,32,33].

#### 2.4.2. Hardness Testing

The PLA reinforced with RHBC composites was tested using a micro-Vickers hardness tester (MC-AT, Fine Spavy Associates & Engineers Pvt. Ltd., Miraj, Maharashtra, India), as per the ASTM standard, to evaluate the hardness. The hardness of the sample was measured three times with a diamond indenter with a dual time of 10 s and a load of 0.05 kg [34]. The hardness is evaluated by measuring the diagonal of the indenter produced by the diamond indenters using Equation (1) [35].

(1) $$\mathrm{Hardness}\;\mathrm{of}\;\mathrm{the}\;\mathrm{sample}\;(\mathrm{HV})=\frac{Lsin(\frac{\emptyset }{2})}{d^{2}}$$

Here, L is the load applied, d is the length of the diagonal for the indenter, and ∅ is the angle of the indenter.

### 2.5. Machine Learning (ML)

Machine learning is an advanced and comprehensive approach for understanding the behavior of composite materials. ML was used for understanding the mechanical behavior of the PLA-reinforced RHBC composites in terms of the complex and non-linear relationship between printing parameters and responses like tensile strength, Young’s modulus, and hardness. The fill pattern printing parameters featured categories encoded using one-hot encoding, where each unique pattern was represented by a binary vector as shown in Table 1. In this study, a multiple linear regression model, a Support Vector Machine model, and a Gaussian process were used for the prediction of responses. The model performance metrics, such as the coefficient of determination (R^2^), mean squared error (MSE), root mean square error (RMSE), and mean absolute error (MAE), obtained with k fold cross validation where k = 3, were used to evaluate the robustness of the model using the following Equations (2)–(5) [36,37].

(2) $$R^{2}=1-\frac{\sum_{i=1}^{a}{{(s}_{i}-{\overset{˘}{s}}_{i})}^{2}}{\sum_{i=1}^{a}{{(s}_{i}-\overset{˘}{s})}^{2}}$$

(3) $$MSE=\frac{1}{a}\sum_{i=1}^{a}{{(s}_{i}-{\overset{˘}{s}}_{i})}^{2}$$

(4) $$RMSE=\sqrt{MSE}=\sqrt{({s_{i}-{\bar{s}}_{i})}^{2}}$$

(5) $$MAE=\frac{1}{a}\sum_{i=1}^{a}\left|s_{i}-{\overset{˘}{s}}_{i}\right|$$

In this context, a is the aggregate number of observations or trials. s_I_—the actual measured value for the i^th^ trial, whereas $\overset{˘}{s}$—the mean of all actual values. Likewise, $\overset{˘}{s_{i}}$—the anticipated value associated with the i^th^ observation.

#### 2.5.1. Multiple Linear Regression (MLR)

A multiple linear regression is an extension of simple linear regression, where the linear relationship of multiple independent variables and the dependent variables is analyzed. This method is employed to predict the targets, such as tensile strength, Young’s modulus, and hardness, by varying the independent variables, such as filler weight %, sample orientation angle, pattern of filling, and nozzle temperature, to targets such as tensile strength, Young’s modulus, and hardness. The dataset with multiple observations was collected from experimental analysis and split into 70% train and 30% test for analyzing the predictive accuracy. The relation is clearly explained in Equation (6) [38].

(6) $$\overset{˘}{s_{i}}=\propto_{0}A+\propto_{1}B+\propto_{2}C+\propto_{3}D$$

Here, $\overset{˘}{s_{i}}$ is a dependent variable or the targets (tensile strength, Young’s modulus, and hardness), and A, B, C, and D are independent variables (filler weight %, sample orientation angle, pattern of filling, and nozzle temperature). $\propto_{0}$ is the intercept of the equation, and $\propto_{1},\propto_{2},\propto_{3}$ are coefficients.

#### 2.5.2. K-Nearest Neighbors (KNN) Regression

K-Nearest Neighbors (KNN) regression was employed to predict the multiple output parameters with the variation in the input printing parameters of the 3D printer. KNN is one of the non-parametric methods most commonly used for prediction, for the given input is the average of its K-Nearest Neighbors in the feature space. To predict the output parameters, the dataset was collected from the experimental data and divided using a 70/30 split, where 70% is for training and 30% for testing, with the number of neighbors as five. The KNN model was trained on the training set and then evaluated on the unseen test set to assess its generalization ability. The analytical relation is shown in Equation (7) [39].

(7) $$\overset{˘}{s_{i}}=\frac{1}{K}\sum_{j}^{k}\overset{˘}{s_{i,j}}$$

Here $\overset{˘}{s_{i,j}}$ is the output of the j-th nearest neighbor.

#### 2.5.3. Gradient Boosting Regression (GBR)

Gradient Boosting Regression (GBR) was employed to predict multiple output parameters based on variations in the input features. GBR is an ensemble learning method that builds a predictive model by combining several weak learners, typically decision trees, in a sequential manner. Each subsequent tree in the model corrects the errors made by the previous one, thereby improving the overall predictive accuracy. The dataset was collected from experimental data and split using a 70/30 ratio, where 70% of the data was used for training and 30% for testing. The GBR model was trained on the training set, and its performance was evaluated on the unseen test set to assess its ability to generalize. The model was trained with 100 estimators (trees) and a learning rate of 0.1, with the goal of minimizing the residual errors iteratively. The final predictions were made by aggregating the results from all the trees. The analytical relation of the GBR model is expressed in Equations (8)–(11) [40].

Define a dataset by

(8) D={((A,B,C,D),si˘)}ni=1

Here  (A, B, C, D) are [filler weight %, sample orientation angle, pattern of filling, and nozzle temperature], and $\overset{˘}{s_{i}}$ is [tensile strength, Young’s modulus, and hardness].

(9) $$S_{n}\left(A,B,C,D\right)=\sum_{n=1}^{n}R_{n}h_{n}\left(A,B,C,D\right)$$

For the gradient descent update

(10) $$S_{n}\left(A,B,C,D\right)=S_{m-1}\left(A,B,C,D\right)+R_{m}h_{m}\left(A,B,C,D\right)$$

Here $R_{n}$ is the learning rate or weight applied to each tree, n is the total number of iterations (trees), and $h_{n}\left(A,B,C,D\right)$ is the $n^{th}$ weak learner (decision tree).

$h_{n}$ is trained to fit the pseudo-residuals as follows:

(11) $$h_{n}\approx \;r_{i}^{n}=\left[\frac{\partial l\left(O_{i},S_{N-1}\left({\left(A,B,C,D\right)}_{i}\right)\right)}{\partial S_{n-1}\left({\left(A,B,C,D\right)}_{i}\right)}\right]$$

Here, $r_{i}^{n}$ is the pseudo-residual for data point i at boosting iteration m. $\overset{˘}{s_{i}}$ is the actual target value, $S_{n-1}\left({\left(A,B,C,D\right)}_{i}\right)$ is the prediction from the model after m−1 iterations, and $\partial l\left(\overset{˘}{s_{i}},S_{n-1}\left({\left(A,B,C,D\right)}_{i}\right)\right)$ are the loss functions (e.g., mean squared error).

#### 2.5.4. Support Vector Machine (SVM)

We predict multiple targets by the variation in the printing feature, including the infill weight % of RHBC/PLA composites. Support Vector Machine (SVM) is a powerful machine learning method used for both classification and regression tasks. In this study, SVM regression was used to find a function that approximates the relationship between printing parameters, including filler weight %, and the output target variables by fitting a function that minimizes the error within specified margin tolerances around the dataset collected from the experimental analysis. The data was split into 70% training and 30% testing. SVM models were trained for each output variable, and predictions were made using the trained models. The predictions for all output variables are shown in the mathematical expressions in Equations (12)–(14) [41].

(12) $$F\left({\left[A,B,C,D\right]}^{T}\right)=w^{T}(A,B,C,D)+b$$

Here [A,B,C,D] is the input vector, w is the weight vector, b is the bias term, and $w^{T}p$ is the dot product between w and (A,B,C,D).

The epsilon-insensitive loss function is

(13) $$L\left(\overset{˘}{s_{i}},\;f\left(\left(A,B,C,D\right)\right)\right)=\left\{\begin{array}{c} 0\;if\;\left|O-f\left(\left(A,B,C,D\right)\right)\right|<€\\ |y-f\left((A,B,C,D)\right)|-€\;other\;wise\; \end{array}\;\right.$$

Here, y is the actual output variable (tensile strength, Young’s modulus, and hardness), $f\left(\left(A,B,C,D\right)\right)$ is the predicted output function, and € is the insensitive margin (tolerance zone).

Objective optimization is defined as

(14) $$\min_{w,b,d,d^{*}}\left(\frac{1}{2}{\left\|w\right\|}^{2}+C\sum_{i=1}^{n}d_{i}+{d^{*}}_{i}\right)$$

Subject to

$$\begin{array}{c} O_{i}-w^{T}{\left(A,B,C,D\right)}_{i}-b\underline{<}€+d_{i}\\ w^{T}{\left(A,B,C,D\right)}_{i}+b-O_{i}\underline{<}€+{d^{*}}_{i}\\ d_{i},{d_{i}}^{*}\underline{>}0 \end{array}$$

Here, $d_{I},{d_{i}}^{*}$—slack variables for positive and negative deviations, €—regularization parameter controlling the penalty of errors, and n—total number of data points.

## 3. Results and Discussion 

### 3.1. Experimental Analysis

#### 3.1.1. Tensile Test

**Figure 4:**
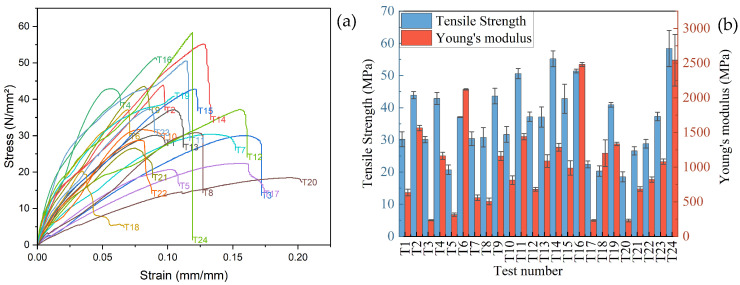
Experimentally obtained (**a**) stress–strain, (**b**) tensile strength, and Young’s modulus for 24 samples.

**Figure 5:**
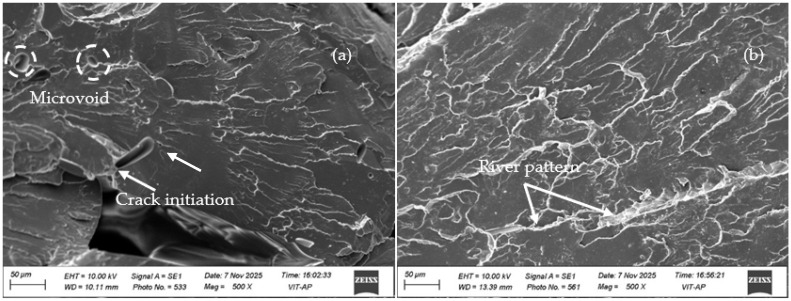
Fractography of (**a**) PLA, (**b**) 20 wt.% RHBC/PLA composites.

The experimental stress–strain results for the 24 test samples are shown in Figure 4a. The results revealed significant variation in stress responses with changes in filler content (wt.%) and other printing parameters, including sample orientation angle, printing pattern, and nozzle temperature. Samples T24, T16, and T14 exhibit the highest stress tolerance, suggesting superior load-bearing capacities. At the same time, samples such as T20, T5, and T17 show lower stress resistance and failure at relatively low stress. Figure 4b illustrates the tensile strength and Young’s modulus results for all the samples and the values are noted in Table 3. It is observed that the tensile strength values vary from 18.5 MPa to 58.5 MPa, with the maximum at T24 and T16, with 58.4 MPa and 51.4 MPa, respectively. The minimum tensile strength is 18.5 MPa at T20 and 20.7 MPa at T5. For Young’s modulus the maximum and minimum are seen at T24 (2542.25 MPa) and T5 (314.34 MPa). These variations showed that the mechanical properties are majorly influenced by the filler content (wt.%) and also influenced by other printing parameters. A higher filler content corresponds to a higher tensile strength and Young’s modulus, suggesting improved material performance, whereas a lower filler content leads to more flexible materials with lower strength and stiffness [42]. To further analyze the effect of RHBC filler in PLA, the SEM fractography of pure PLA and 20 wt. % RHBC/PLA composites at the same conditions are analyzed, i.e., T2 and T24 as shown in Figure 5. Figure 5a shows that the tensile fracture mechanism of pure PLA typically involves the formation of microvoids due to stress concentration, which expand as the polymer chains break apart. As the stress increases, cracks initiate at these voids, propagating along the material until failure occurs. These characteristics indicate the material’s brittle nature [43]. In contrast, the fractography of the 20% RHBC/PLA composite in Figure 5b shows a river pattern, which is characteristic of severe brittle behavior [44].

#### 3.1.2. Hardness

**Figure 6:**
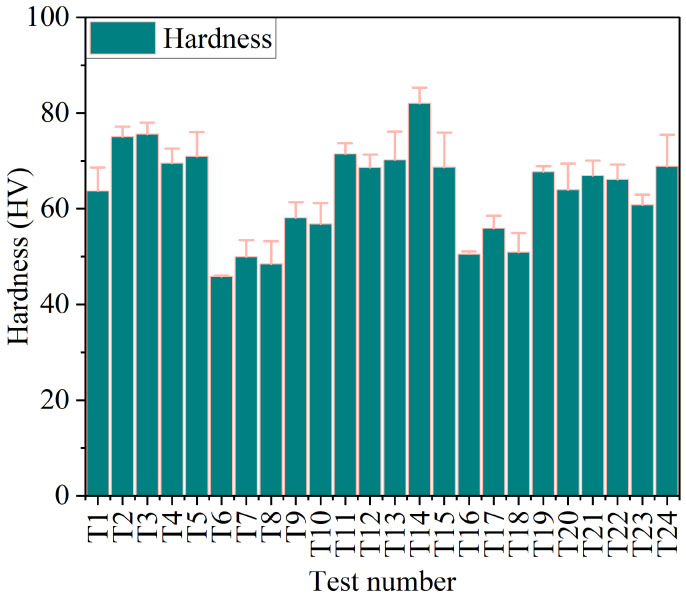
Hardness distribution for 24 tests for all samples.

Figure 6 shows the experimentally obtained Vickers hardness (HV) results for all 24 tests, and the values are presented in Table 3. It was observed that the values of hardness are between 45.85 HV and 82 HV. The maximum hardness obtained at test T13 was 82 HV, while the minimum hardness obtained at test T6 was approximately 45.85 HV. The results suggest that the most influential factor on hardness is filler content, and variations in it significantly affect hardness values. The values obtained across the tests, such as 75.08 HV (T2), 75.57 HV (T3), and 70.95 HV (T5), suggest that hardness varies with test conditions. Some tests showed higher hardness than others, indicating varying influences on the material’s properties.

### 3.2. Analysis of Variance (ANOVA)

#### 3.2.1. Tensile Strength

Table 4 shows the ANOVA results for the tensile strength of all the samples. The results indicate that the overall model is statistically significant, with an F-value of 23.69 and a *p*-value < 0.0001. The results also confirm that there is a large portion of the variability in tensile strength. It was noted that filler content (A) has a significant effect with a *p*-value of 0.0001. Besides this, the orientation angle (B) and nozzle temperature (D) also have significant effects and noted *p*-values of 0.0054 and 0.0031, respectively. Compared with other parameters, the fill pattern (C) has an insignificant effect, with a *p*-value of 0.4718. The interactions, such as filler content and orientation angle (AB) with *p* = 0.0999, and filler content and nozzle temperature (A^2^D^2^) with *p* = 0.0489, show combined effects that influence tensile strength. Higher-order interactions, such as A^2^ (*p* < 0.0001), A^2^B (*p* = 0.0003), and C^2^D^2^ (*p* = 0.0228), significantly affect the material’s tensile strength, underscoring the importance of the non-linear and combined effects of these factors. The R^2^ value of 0.9685 and the adjusted R^2^ value of 0.9277 indicate that the model fits the data well, explaining 96.85% of the variability. Equation (15) is used to predict tensile strength as a function of these factors.

**Figure 7:**
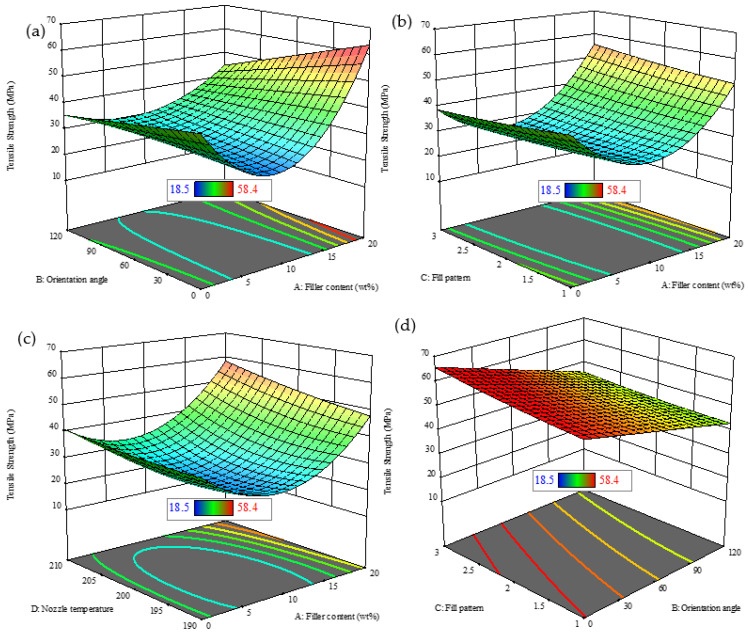
Three-dimensional plots for tensile strength with varying factors. (**a**) Orientation angle vs. filler content; (**b**) fill pattern vs. filler content; (**c**) nozzle temperature vs. filler content; (**d**) fill pattern vs. orientation angle.

Figure 7a–d present a comprehensive 3D evaluation of tensile strength influenced by various factors. Figure 7a demonstrates that at an angle between 0° and 30°, there is an enhancement of tensile strength as filler content is raised. However, at an orientation angle between 0° and 30°, higher values begin to reduce tensile strength. Figure 7b highlights that both filler content and fill pattern significantly affect tensile strength. The optimal combinations of these two factors lead to the highest tensile strength. In Figure 7c, nozzle temperature is shown to have a key impact, with tensile strength peaking at temperatures between 190 and 200 °C, especially when combined with a higher filler content. Figure 7d indicates that fill pattern and orientation angle also influence tensile strength, but their impact is less significant than that of filler content and nozzle temperature. The results show that the filler content is the dominant factor affecting tensile strength, with nozzle temperature and fill pattern playing supporting roles.



Tensile strength = 10.719 − 17.181(A) − 0.017500(B) − 17.95841(C) − 0.288715(D) + 0.028083(AB) + 0.252500(AC) + 0.060136(AD) − 0.019167(BC) + 0.475563(A^2^) 0.001629(A^2^B) + 0.000268(CD^2^) − 5.07948E−06(A^2^D^2^) + 0.000045(C^2^D^2^)
                        (15)


#### 3.2.2. Young’s Modulus

The ANOVA results for the Young’s modulus of all 24 tests are presented in Table 5. The results show that the overall model is statistically significant with an F-value of 20.61 and a *p*-value of 0.0017, explaining a large portion of the variability in Young’s modulus. Filler content (A) has a marginally significant effect with a *p*-value of 0.0589, while orientation angle (B), fill pattern (C), and nozzle temperature (D) have insignificant effects, with *p*-values of 0.7052, 0.3299, and 0.1295, respectively. Significant interactions such as filler content and orientation angle (AC) with *p* = 0.0178, filler content and nozzle temperature (AD) with *p* = 0.0056, and fill pattern and nozzle temperature (C^2^D) with *p* = 0.0057 influence the response. The higher-order interactions, such as A^2^ with *p* < 0.0001, A^2^B with *p* = 0.0026, and A^2^C with *p* = 0.0159, significantly impact Young’s modulus, indicating that the non-linear and combined effects of these factors play an essential role. The R^2^ value of 0.9867 and the adjusted R^2^ value of 0.9388 suggest that the model fits the data very well, explaining 98.67% of the variability. Equation (16) is used to predict Young’s modulus based on the identified factors and interactions.

**Figure 8:**
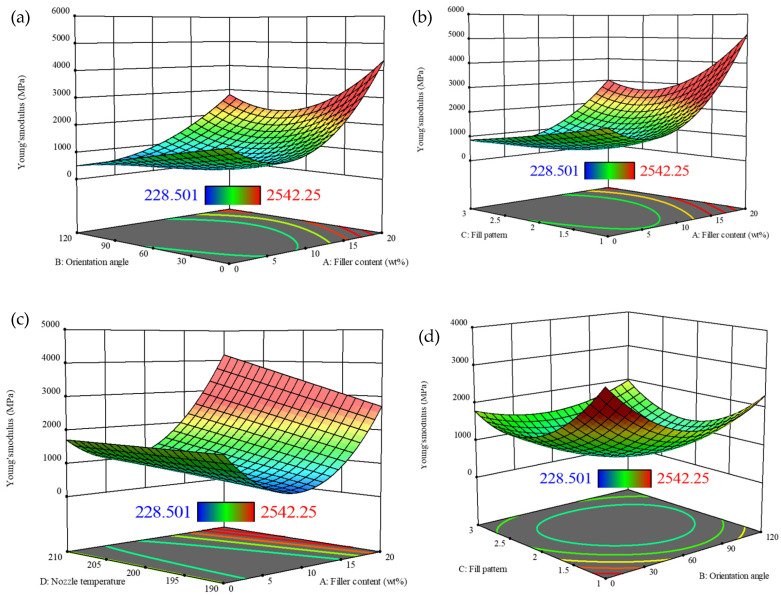
Three-dimensional plots for Young’s modulus with varying factors. (**a**) Orientation angle vs. filler content; (**b**) fill pattern vs. filler content; (**c**) nozzle temperature vs. filler content; (**d**) fill pattern vs. orientation angle.

Figure 8 presents a comprehensive 3D evaluation of Young’s modulus, examining the influence of filler content, orientation angle, fill pattern, and nozzle temperature. In Figure 8a, Young’s modulus increases significantly from 228.5 MPa to 2542.25 MPa as filler content rises, especially when the orientation angle is between 0° and 30°, beyond which further increases in orientation angle reduce Young’s modulus. Figure 8b shows that both filler content and fill pattern affect Young’s modulus, with the highest modulus values observed at around 20 wt.% filler content and specific fill patterns. Figure 8c shows that nozzle temperature significantly affects Young’s modulus, peaking between 190 °C and 200 °C, especially at a higher filler content. Finally, Figure 8d indicates that, while fill pattern and orientation angle also influence Young’s modulus, their effects are secondary to those of filler content and nozzle temperature. The results show that the filler content is the dominant factor affecting Young’s modulus, with nozzle temperature and fill pattern playing supporting roles.

Young’s modulus = 483.25 − 195.53(A) + 22.73(B) + 86.57(C) − 145.50(D) − 80.66(AB) − 278.44(AC) + 372.07(AD) + 266.88(BC) + 930.88(A^2^) + 93.19(B^2^) + 105.54(C^2^) − 545.68(A^2^B) − 405.71(A^2^C) + 603.64(AB^2^) + 578.28(AC^2^) − 263.57(B^2^C) + 309.84(B^2^D) + 525.39(C^2^D) (16)

#### 3.2.3. Hardness

The ANOVA results for the hardness values of all 24 tests are presented in Table 6. The results show that the overall model is statistically significant with an F-value of 14.03 and a *p*-value of 0.0004. The results show that orientation angle (B) and fill pattern (C) have substantial effects on hardness, with *p*-values of 0.0121 and 0.0002, respectively. However, filler content (A) and nozzle temperature (D) have marginal or insignificant effects with *p*-values of 0.0609 and 0.2535, respectively. Besides this, significant interactions such as filler content and orientation angle (AC) with *p* = 0.0017, filler content and nozzle temperature (AD) with *p* = 0.0086, and fill pattern and nozzle temperature (CD) with *p*-value of 0.0313 are noted. Higher-order interactions, such as B^2^ (*p* = 0.0033), A^2^D (*p* = 0.0216), AB^2^ (*p* = 0.0464), and A^2^B^2^ (*p* = 0.0004), also significantly affect hardness, indicating that the combined and non-linear effects of these factors play a crucial role in determining the material’s hardness. The R^2^ value of 0.9634 and the adjusted R^2^ value of 0.8947 suggest that the model fits the data well, explaining 96.34% of the variability. Equation (17) is used to predict hardness based on the identified factors and interactions.

**Figure 9:**
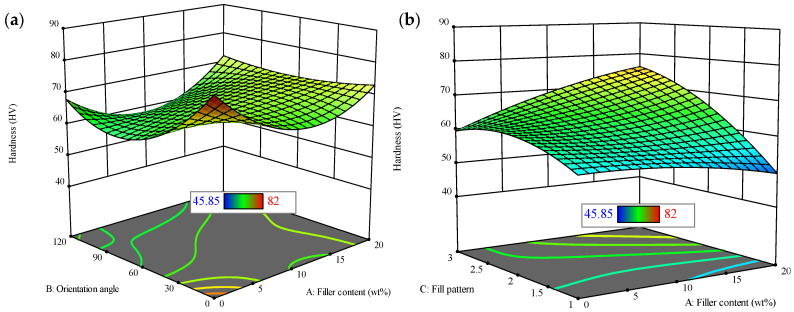

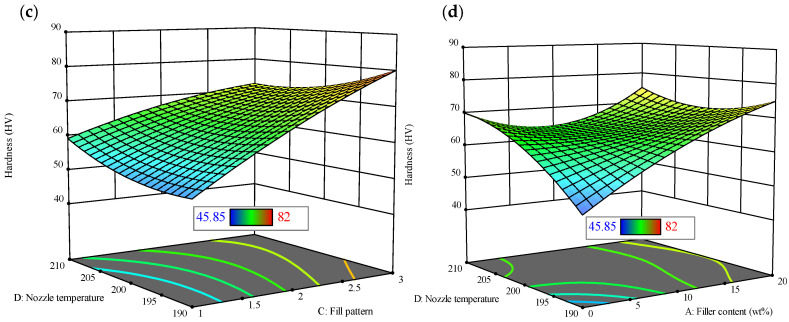
Three-dimensional plots for hardness with varying factors. (**a**) Orientation angle vs. filler content; (**b**) fill pattern vs. filler content; (**c**) nozzle temperature vs. filler content; (**d**) fill pattern vs. orientation angle.

Figure 9 presents a comprehensive 3D evaluation of hardness values (HVs), focusing on the influence of filler content, orientation angle, fill pattern, and nozzle temperature. Figure 9a shows that the hardness values ranges from 45.85 to 82 HV. The HV increases significantly with higher filler content, especially when the orientation angle is between 0° and 30°. However, when the orientation angle exceeds 30°, hardness begins to decrease despite a higher filler content. Figure 9b shows that specific fill patterns, combined with an increased filler content (up to 20 wt.%), yield the highest hardness values. At lower filler contents, specific fill patterns result in reduced hardness. In Figure 9c, the nozzle temperature is shown to play a crucial role in hardness, with the highest hardness value of 82 HV occurring at temperatures between 190 °C and 200 °C and at a higher filler content. Figure 9d indicates that while both fill pattern and orientation angle influence hardness, their effects are secondary to those of filler content and nozzle temperature. The results suggest that a higher filler content (up to 20 wt.%) is the dominant factor affecting hardness, with nozzle temperature and fill pattern playing supporting roles in optimizing the material’s properties.

Hardness = 65.42 + 1.67(A) − 3.49(B) + 6.29(C) + 1.36(D) + 3.06(AB) + 5.47(AC) − 5.39(AD) − 4.07(CD) − 6.46(B^2^) + 5.44(A^2^D) − 5.19(AB^2^) + 4.35(AD^2^) + 3.33(B^2^C) + 12.83(A^2^B^2^) − 4.29(A^2^C^2^) (17)

### 3.3. Machine Learning 

#### Tensile Strength

**Figure 10:**
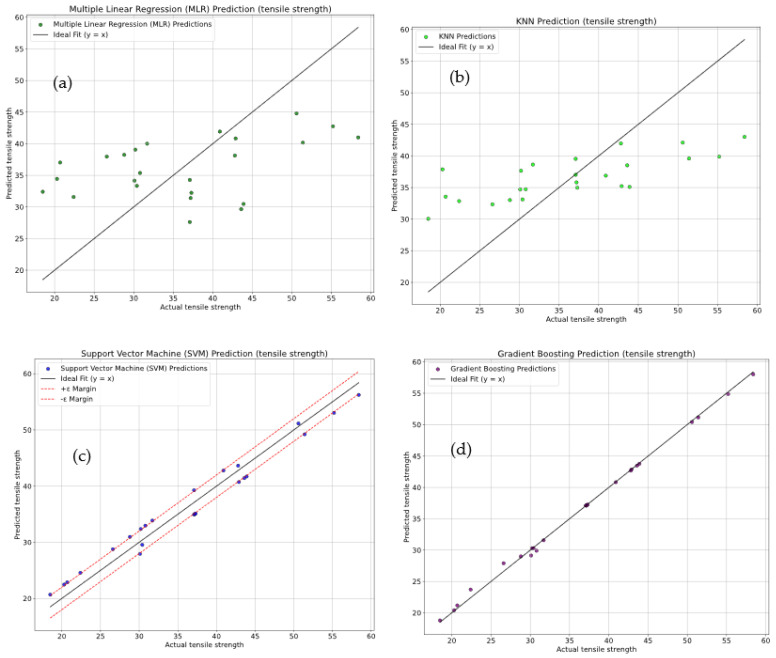
Sector plots for comparison of actual vs. predicted tensile strength using (**a**) multiple linear regression (MLR), (**b**) K-Nearest Neighbors (KNN) Regression, (**c**) Support Vector Machine (SVM), (**d**) Gradient Boosting.

Figure 10a–d show a comparison plot between actual versus predicted tensile strength values. There are four regression models used in this analysis: (i) multiple linear regression (MLR), (ii) K-Nearest Neighbors (KNN), (iii) Support Vector Machine (SVM), and (iv) Gradient Boosting. Figure 10a shows that the MLR model has a significant scatter around the ideal line (y = x), indicating a poor fit and limited predictive accuracy. The evaluation metrics for MLR reveal an R^2^ of 17.29 ± 4.6%, along with high error metrics, MSE = 97.20 ± 4.1%, RMSE = 9.86 ± 3.8%, and MAE = 8.68 ± 4.3%. These results indicate a substantial prediction error. Figure 10b confirms that the KNN model improves upon MLR but still shows considerable scatter, with an R^2^ of 36.53 ± 4.2%, MSE of 74.59 ± 3.9%, RMSE of 8.64 ± 4.5%, and MAE of 7.15 ± 3.7%. These results indicate that its predictions are less precise than expected. Figure 10c shows that the SVM model performs better, with an R^2^ of 96.48 ± 3.4% with reduced error values of MSE = 4.14 ± 3.1%, RMSE = 2.04 ± 3.6%, and MAE = 1.98 ± 4.0%. Figure 10d demonstrates that Gradient Boosting is the most accurate model when evaluated on the test dataset. In this model, the values of R^2^ of 97.79 ± 3.8% and minimal error metrics MSE = 0.246 ± 4.5%, RMSE = 0.496 ± 3.9%, and MAE = 0.313 ± 4.2% are observed. This model clearly outperforms all others in terms of prediction accuracy and minimal errors. The results demonstrated that Gradient Boosting is the best model for predicting tensile strength, offering the most reliable and precise predictions on unseen test data.

Supplementary Figure S1a–d shows a comparison plot of the tensile strength classification for all the models through confusion matrix. The tensile strength values were categorized into four classes—Brittle, Semi-Brittle, Elastic, and Highly Elastic—based on predefined tensile strength range thresholds derived from the experimental dataset, which are determined by strain at break criteria. This analysis was carried out via various machine learning models, multiple linear regression (MLR), K-Nearest Neighbors (KNN), Support Vector Machine (SVM), and Gradient Boosting. As shown in Supplementary Figure S1a, the MLR model demonstrated the weakest performance, achieving an accuracy of 33.33%. The model struggled to classify the Brittle, Semi-Brittle, and Elastic categories, with frequent misclassifications, particularly between Brittle and Semi-Brittle. It has low recall values for Brittle (0%) and Highly Elastic (25%). In contrast, Supplementary Figure S1b shows that the KNN model has an improved accuracy of 54.17% and a recall of 80% for Highly Elastic. However, it still struggled with Semi-Brittle, which had a lower recall of 57%, and misclassified Elastic in some instances. Supplementary Figure S1c shows that the SVM model has a stronger performance, with an accuracy of 70.83%, and achieved a recall of 100% for Brittle and Highly Elastic. But it had a lower recall for Semi-Brittle and Elastic. Among all ML models, the Gradient Boosting model in Supplementary Figure S1d outperformed all other models, achieving an 83.33% accuracy with strong recall values for Brittle (85.7%) and Highly Elastic (85.7%). It showed a good balance across all classes, particularly for Brittle and Highly Elastic, making it the most effective model for tensile strength classification.

Supplementary Figure S2 presents a detailed comparison of the tensile strength classification of all samples using SHAP values. SHAP is used to interpret the impact of various features across the four machine learning models, multiple linear regression, K-Nearest Neighbors, Support Vector Machine, and Gradient Boosting. As shown in Supplementary Figure S2a,b, the MLR model demonstrated that filler content has the highest impact, with the SHAP value range spanning from −0.75 to 0.75, followed by nozzle temperature with a range of −0.5 to 0.5 and orientation angle with a range of −0.25 to 0.25. Fill pattern has the least influence, with values around 0.05. As shown in Supplementary Figure S2c,d, the KNN model demonstrated that the filler content again shows the most significant impact, with a SHAP range from −1.5 to 1.5, followed by nozzle temperature, ranging from −0.5 to 0.5, and orientation angle, with a range of −0.25 to 0.25. Fill pattern continues to have a minimal effect. As shown in Supplementary Figure S2e,f for the SVM model, filler content dominates, with a SHAP value range from −0.75 to 0.75, followed by nozzle temperature with a range of −0.5 to 0.5 and orientation angle with a range of −0.25 to 0.25, while fill pattern shows negligible influence. As shown in Supplementary Figure S2g,h for the Gradient Boosting model, filler content has the widest SHAP value range from −15 to 15, with nozzle temperature ranging from −3 to 3 and orientation angle from −2 to 2, showing moderate impact. The fill pattern again shows the least influence. Across all models, filler content is the most influential feature for predicting tensile strength, with the highest impact and widest SHAP value range, particularly in Gradient Boosting. Nozzle temperature and orientation angle follow as secondary features, while fill pattern consistently shows minimal influence. These results demonstrate that filler content plays a central role in determining the tensile strength of all samples, with Gradient Boosting providing the best performance, followed by SVM, while KNN and MLR show lower accuracies.

**Figure 11:**
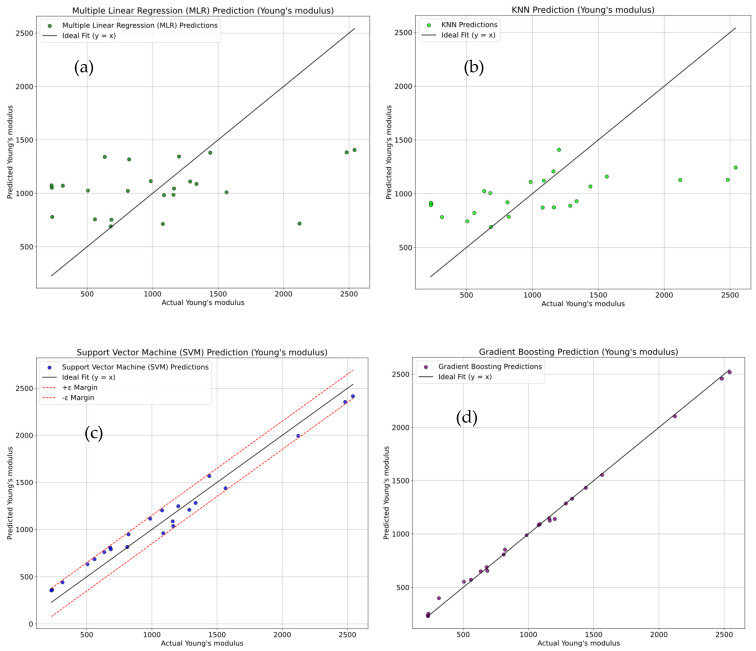
Sector plots for comparison of actual vs. predicted Young’s modulus using (**a**) multiple linear regression (MLR), (**b**) K-Nearest Neighbors (KNN) Regression, (**c**) Support Vector Machine (SVM), (**d**) Gradient Boosting.

Figure 11a–d show the sector plots that compare the actual versus predicted Young’s modulus. The analysis is performed via four regression models, multiple linear regression (MLR), K-Nearest Neighbors (KNN), Support Vector Machine (SVM), and Gradient Boosting. As shown in Figure 11a, the MLR model exhibits significant scatter about the ideal line (y = x), which indicates a poor fit and less accurate predictions. The evaluation metrics for MLR show an R^2^ of 12.52 ± 0.88% and high error metrics (MSE = 352,752 ± 24,693, RMSE = 593.93 ± 41.58, and MAE = 453.65 ± 31.76). These results indicate a high prediction error. As shown in Figure 11b, the KNN model shows a moderate improvement, but the points still exhibit noticeable spread, with an R^2^ of 24.65 ± 1.73%, with high error values (MSE = 303,866 ± 21,271, RMSE = 551.24 ± 38.59, and MAE = 416.04 ± 29.12). As shown in Figure 11c, the SVM model performs better, with a high R^2^ of 96.72 ± 6.77% with significantly reduced errors (MSE = 13,227 ± 926, RMSE = 115.01 ± 8.05, and MAE = 110.17 ± 7.71). These results demonstrated a stronger fit. Figure 11d shows that the Gradient Boosting model provides the best predictions, with an impressive R^2^ of 98.79 ± 6.92% with very low error metrics (MSE = 817.49 ± 57.22, RMSE = 28.59 ± 2.00, and MAE = 19.91 ± 1.39). This model outperforms among all others in terms of predictive accuracy and error minimization.

Supplementary Figure S3a–d shows the comparison plot for the Young’s modulus classification of all tested samples. The Young’s modulus values in the experimental data set were categorized into four classes: Brittle, Semi-Brittle, Elastic, and Highly Elastic. The analysis is performed through various machine learning models, including multiple linear regression (MLR), K-Nearest Neighbors, Support Vector Machine, and Gradient Boosting. Supplementary Figure S3a shows that the MLR model demonstrated the weakest performance, achieving an accuracy of 33.33%. It struggled to classify the Brittle, Semi-Brittle, and Elastic categories, with frequent misclassifications, particularly between Brittle and Semi-Brittle.

It also showed low recall values for Brittle (0%) and Highly Elastic (25%). In contrast, the KNN model, as shown in Supplementary Figure S3b, demonstrated an improved accuracy of 45.83% and performed well with a recall of 83.3% for Highly Elastic. However, it still struggled with Semi-Brittle, which had a lower recall of 57%, and misclassified Elastic in some instances. The SVM model shown in Supplementary Figure S3c demonstrated a stronger performance, with an accuracy of 79.17%, and achieved a recall of 100% for Brittle and Highly Elastic. But it had a lower recall for Semi-Brittle and Elastic. Among all models, the Gradient Boosting model shown in Supplementary Figure S3d outperformed all other models, achieving 100% accuracy with strong recall values for Brittle (100%) and Highly Elastic (100%). It showed a good balance across all classes, particularly for Brittle and Highly Elastic, making it the most effective model for tensile strength classification.

Supplementary Figure S4a–h presents a detailed comparison of the SHAP and feature impacts on Young’s modulus predictions for RHBC/PLA composites using four machine learning models, multiple linear regression (MLR), K-Nearest Neighbors (KNN), Support Vector Machine (SVM), and Gradient Boosting. As shown in Supplementary Figure S4a,b for multiple linear regression, filler content exhibits the most significant influence on the model (Supplementary Figure S4b), with SHAP values (Supplementary Figure S4a) ranging from −0.2 to 0.3, followed by orientation angle (−0.1 to 0.1) and nozzle temperature (−0.1 to 0.1). The fill pattern shows the least impact, with values near 0. As shown in Supplementary Figure S4c,d for the KNN model, filler content is the most influential factor (Supplementary Figure S4d). Supplementary Figure S4c, indicating a significant impact on SHAP values range from −100 to 200prediction, followed by orientation angle (−0.1 to 0.1) and nozzle temperature (−0.2 to 0.1). Supplementary Figure S4e,f shows the SVM model, where filler content has the most significant influence (Supplementary Figure S4f), with SHAP values (Supplementary Figure S4e) ranging from −0.75 to 0.75, followed by nozzle temperature (−0.25 to 0.25) and orientation angle (−0.25 to 0.25). As shown in Supplementary Figure S4g,h for the Gradient Boosting model, the filler content has the most significant factor (Supplementary Figure S4h), with the widest SHAP value (Supplementary Figure S4g) range, from −800 to 600, indicating its dominant role in predicting Young’s modulus, followed by orientation angle (−0.2 to 0.2) and nozzle temperature (−0.2 to 0.2). The results also demonstrate that the fill pattern has the least influence across all models.

**Figure 12:**
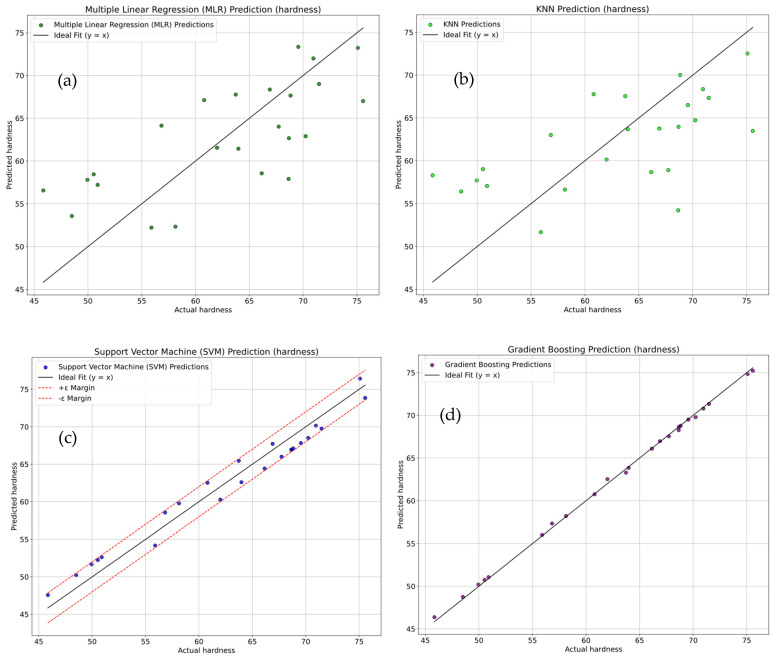
Sector plots for comparison of actual vs. predicted hardness using (**a**) multiple linear regression (MLR), (**b**) K-Nearest Neighbors (KNN) Regression, (**c**) Support Vector Machine (SVM), and (**d**) Gradient Boosting.

The sector plots shown in Figure 12a–d compare the actual versus predicted hardness values using four regression models, multiple linear regression (MLR), K-Nearest Neighbors (KNN), Support Vector Machine (SVM), and Gradient Boosting. The MLR model shown in Figure 12a, demonstrated a moderate fit with an R^2^ of 52.32 ± 2.62%, accompanied by relatively high errors (MSE = 35.26 ± 1.76, RMSE = 5.94 ± 0.30, MAE = 5.16 ± 0.26), indicating reasonable but not optimal predictive accuracy. The KNN model shown in Figure 12b showed R^2^ of 37.76 ± 1.89% and higher error values (MSE = 46.03 ± 2.30, RMSE = 6.78 ± 0.34, MAE = 5.72 ± 0.29). SVM shown in Figure 12c offers better performance with a high R^2^ of 96.39 ± 4.82% with lower errors (MSE = 2.67 ± 0.13, RMSE = 1.63 ± 0.08, MAE = 1.61 ± 0.08), but still falls short of the top performer. Among all ML model, Gradient Boosting shown in Figure 12d outperforms among all ML models, with an impressive R^2^ of 96.80 ± 4.84% and minimal prediction errors (MSE = 0.077 ± 0.004, RMSE = 0.278 ± 0.014, MAE = 0.226 ± 0.011).

Supplementary Figure S5a–d shows a detailed comparison of the hardness classification of all samples using various machine learning models, multiple linear regression (MLR), K-Nearest Neighbors (KNN), Support Vector Machine (SV), and Gradient Boosting. Here, the experimental dataset was split into four categories like Brittle, Semi-Brittle, Elastic, and Highly Elastic, as per the predefined dataset. The MLR model, shown in Supplementary Figure S5a, demonstrated the weakest performance, achieving an accuracy of 45.83%. It struggled to classify the Brittle, Semi-Brittle, and Elastic categories, with frequent misclassifications, particularly between Brittle and Semi-Brittle, and low recall values for Brittle (0%) and Highly Elastic (25%). In contrast, the KNN model shown in Supplementary Figure S5b showed improved accuracy of 29.17% and performed well with a recall of 83.3% for Highly Elastic. Nevertheless, it still struggled with Semi-Brittle, which had a lower recall of 57%. It misclassified Elastic in some instances. Supplementary Figure S5c shows for SVM model, which demonstrated a stronger performance. Here, the accuracy seen is 83.33%, and achieved a recall of 100% for Brittle and Highly Elastic. However, it had a lower recall for Semi-Brittle and Elastic. Supplementary Figure S5d for the Gradient Boosting model, showed a best performance model, where it outperformed compare to all other models. It achieved close to 100% accuracy with strong recall values for Brittle (100%) and Highly Elastic (100%). It showed a good balance across all classes, particularly for Brittle and Highly Elastic, making it the most effective model for tensile strength classification.

Supplementary Figure S6a–h presents a detailed comparison of the hardness classification of all samples using SHAP values across four machine learning models, multiple linear regression (MLR), K-Nearest Neighbors (KNN), Support Vector Machine (SVM), and Gradient Boosting. In the multiple linear regression shown in Supplementary Figure S6a,b, the fill pattern has the largest impact, with a SHAP value range from −0.8 to 0.8, followed by orientation angle (−0.6 to 0.6) and nozzle temperature (−0.4 to 0.4). However, it showed that filler content has the least influence. The average SHAP values show that the fill pattern has the highest impact (0.35), followed by orientation angle (0.15) and nozzle temperature (0.12). As shown in Supplementary Figure S6c,d for the KNN model, the fill pattern remains the dominant feature, with a SHAP range from −6 to 6, followed by orientation angle (−2 to 2) and nozzle temperature (−1 to 1). The average SHAP values for fill pattern (2.5), orientation angle (1.0), and nozzle temperature (0.5) confirm its prominence. Supplementary Figure S6e,f show the SVM model, demonstrating that the fill pattern has the most significant impact with the SHAP values ranged from −1 to 1, followed by the orientation angle (−0.5 to 0.5) and filler content (−0.25 to 0.25). The average SHAP value for the fill pattern is 0.3, followed by the orientation angle (0.2) and filler content (0.1). Supplementary Figure S6g,h show for Gradient Boosting, where the fill pattern shows the widest SHAP value range from −12 to 7. The results indicate the strongest influence on the model’s output, followed by nozzle temperature (−3 to 3) and orientation angle (−2 to 2). It is also noted that the filler content has the least effect. The average SHAP values for fill pattern (3.5), nozzle temperature (1.5), and orientation angle (1.0) demonstrated the prominence of fill pattern. Across all models, fill pattern consistently shows the highest impact on predicting hardness, followed by orientation angle and nozzle temperature, with filler content showing minimal influence. These results demonstrate that the fill pattern is the most influential feature in predicting hardness.

### 3.4. Correlation Heatmap 

**Figure 13:**
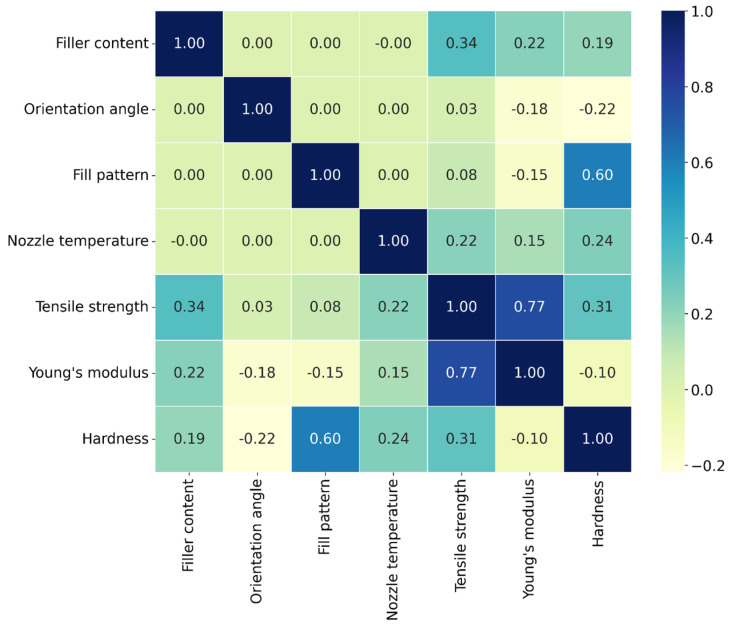
Correlation heatmap between the factors and responses.

Figure 13 shows the correlation heatmap results, which provide the relation between printing parameters, such as orientation angle, fill pattern, nozzle temperature, and filler content, across the responses, including tensile strength, Young’s modulus, and hardness. The matrix shows the strength and direction of relationships between pairs of variables, ranging from −1 to 1. Specifically, filler content has a moderate positive correlation with tensile strength (0.34) and a weaker positive correlation with Young’s modulus (0.22). It has a weak positive correlation with hardness (0.19) and negligible correlations with orientation angle (−0.00), fill pattern (−0.00), and nozzle temperature (−0.00). Orientation angle shows a weak correlation with the mechanical properties, namely tensile strength (0.03), Young’s modulus (−0.18), and hardness (−0.22). Fill pattern has a moderate positive correlation with hardness (0.60) and weaker correlations with tensile strength (0.08), Young’s modulus (−0.15), and nozzle temperature (0.00). Nozzle temperature has moderate positive correlations with tensile strength (0.22), Young’s modulus (0.15), and hardness (0.24), with very weak correlations with other parameters. Tensile strength and Young’s modulus show a strong positive correlation (0.77), with tensile strength also correlating moderately with hardness (0.31). Young’s modulus has a weak negative correlation with hardness (−0.10), while hardness correlates most strongly with fill pattern (0.60) and moderately with tensile strength (0.31) and nozzle temperature (0.24). This matrix provides insights into how these material properties interrelate, which is useful for optimization and predicting material performance.

### 3.5. Model Prediction Comparison

**Figure 14:**
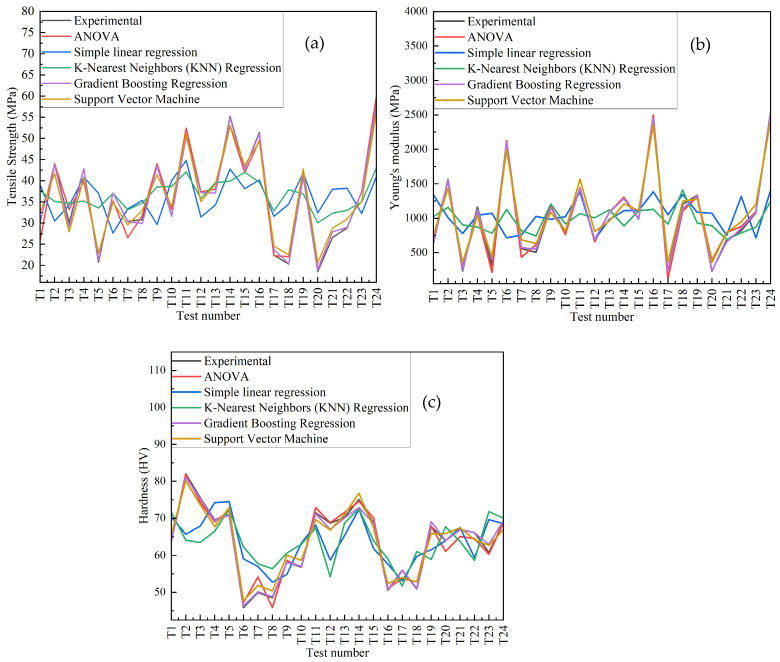
Comparison of experimental and prediction results for all samples: (**a**) tensile strength, (**b**) Young’s modulus, (**c**) hardness.

Figure 14 shows the comparison plot between the experimental and model predictions, such as ANOVA and machine learning models of all tested samples. The properties considered are tensile strength (MPa), Young’s modulus (MPa), and hardness (HV). As shown in Figure 14a, the tensile strength obtained from machine learning models, Gradient Boosting and SVM, and ANOVA aligns most closely with the experimental data. Here, Gradient Boosting provides the most accurate predictions. SVM is consistent but shows minor deviations. MLR and KNN show significantly larger deviations from the experimental pattern. As shown in Figure 14b, the Young’s modulus predicted by Gradient Boosting, SVM, and ANOVA continues to demonstrate a strong predictive performance, closely following the experimental data. Meanwhile MLR and KNN show larger deviations from the experimental data. As shown in Figure 14c, the hardness predicted by Gradient Boosting, SVM, and ANOVA once again shows the best performance, with predictions closely matching the experimental values. Multiple linear regression and KNN show larger discrepancies. Across all three properties (the results are summarized in Table 7) Gradient Boosting performs the best, with Gradient Boosting emerging as the top model due to its ability to consistently track the experimental data, followed by SVM, which is a reliable model but slightly less accurate than the former two.

## 4. Conclusions 

This study focuses on the optimization and prediction of rice husk biochar (RHBC) as a sustainable reinforcement in polylactic acid (PLA) using a 3D printing route. The influence of 3D printing parameters such as filler content (0 wt.%, 10 wt.%, 20 wt.%), nozzle temperature (190 °C, 200 °C, 210 °C), orientation angle (0°, 60°, 120°), and fill pattern (hexagon, triangle, and 3D infill) were systematically analyzed. Additionally, machine learning models (i.e., MLR, KNN, SVM, and Gradient Boosting) were used for prediction. The important results are summarized as follows:

- Tensile strength, Young’s modulus, and hardness showed considerable variation with variation in filler content and other printing parameters.
- ANOVA identified filler content as the most significant parameter influencing tensile strength, Young’s modulus, orientation angle, and fill pattern for hardness.
- Among machine learning models, Gradient Boosting provided the best predictive accuracy with R^2^ values of 97.79% for tensile strength, 98.79% for Young’s modulus, and 96.8% for hardness.
- SHAP and feature importance analysis confirmed that filler content, nozzle temperature, and orientation angle were the key factors influencing the material’s mechanical properties.

## Figures and Tables

**Table 1:** Factors and levels for experimental design.

S. No	Factors	Lower	Middle	Higher
1	Filler content (wt.%)	0 (pure PLA)	10	20
2	Sample orientation angle (^o^)	0 (linear direction)	60	120
3	Pattern of filling	−1 (hexagon)	0 (triangle)	+1 (3D infill)
4	Nozzle temperature (°C)	190	200	210

**Table 2:** Experimental design.

Test Number	A: Filler Content (wt. %)	B: Orientation Angle	C: Fill Pattern	D: Nozzle Temperature
T1	10	0	Triangle	210
T2	0	0	Triangle	200
T3	10	60	3D infill	190
T4	10	60	3D infill	210
T5	10	0	3D infill	200
T6	0	60	Triangle	190
T7	10	120	Triangle	190
T8	10	120	Hexagon	200
T9	0	60	Hexagon	200
T10	10	120	Triangle	210
T11	20	60	Triangle	210
T12	0	120	Triangle	200
T13	0	60	Triangle	210
T14	20	60	3D infill	200
T15	20	60	Triangle	190
T16	20	60	Hexagon	200
T17	10	60	Hexagon	190
T18	10	0	Hexagon	200
T19	20	120	Triangle	200
T20	10	0	Triangle	190
T21	10	120	3D infill	200
T22	10	60	Hexagon	210
T23	0	60	3D infill	200
T24	20	0	Triangle	200

**Table 3:** Experimental results for RHBC/PLA composites.

Test Number	Tensile Strength	Young’s Modulus	Hardness
T1	30.2	634.4535	63.725
T2	43.9	1564.719	75.075
T3	30.1	233.737	75.5667
T4	42.9	1163.413	69.55
T5	20.7	314.344	70.95
T6	37.1	2122.087	45.85
T7	30.4	559.5106	49.95
T8	30.8	505.5404	48.5
T9	43.6	1159.541	58.125
T10	31.7	810.9274	56.825
T11	50.6	1440.029	71.475
T12	37.2	680.1563	68.65
T13	37.1	1086.955	70.225
T14	55.2	1286.746	82
T15	42.8	986.8847	68.675
T16	51.4	2481.906	50.525
T17	22.4	230.4954	55.9
T18	20.3	1202.114	50.9
T19	40.9	1335.049	67.725
T20	18.5	228.5005	63.975
T21	26.6	685.3069	66.9
T22	28.8	820.3884	66.15
T23	37.3	1078.132	60.8
T24	58.4	2542.252	68.825

**Table 4:** Analysis of variance summary of tensile strength based on varying factors.

Source	Sum of Squares	df	Mean Square	F-Value	*p*-Value
Model	2731.91	13	210.15	23.69	<0.0001
A—Filler content (wt.%)	331.80	1	331.80	37.40	0.0001
B—Orientation angle (°)	111.01	1	111.01	12.51	0.0054
C—Fill pattern	4.96	1	4.96	0.5592	0.4718
D—Nozzle temperature (°C)	133.33	1	133.33	15.03	0.0031
AB	29.16	1	29.16	3.29	0.0999
AC	25.50	1	25.50	2.87	0.1208
AD	15.21	1	15.21	1.71	0.2197
BC	5.29	1	5.29	0.5963	0.4579
A^2^	1574.11	1	1574.11	177.43	<0.0001
A^2^B	254.80	1	254.80	28.72	0.0003
CD^2^	103.75	1	103.75	11.69	0.0066
A^2^D^2^	44.55	1	44.55	5.02	0.0489
C^2^D^2^	64.03	1	64.03	7.22	0.0228
Residual	88.72	10	8.87		
Cor Total	2820.63	23			
R^2^ = 0.9685, Adjacent R^2^ = 0.9277					

**Table 5:** Analysis of variance summary of Young’s modulus based on varying factors.

Source	Sum of Squares	df	Mean Square	F-Value	*p*-Value
Model	9.549 × 10^6^	18	5.305 × 10^5^	20.61	0.0017
A—Filler Content	1.529 × 10^5^	1	1.529 × 10^5^	5.94	0.0589
B—Orientation angle	4134.73	1	4134.73	0.1606	0.7052
C—Fill pattern	29,975.13	1	29,975.13	1.16	0.3299
D—Nozzle temperature	84,677.31	1	84,677.31	3.29	0.1295
AB	26,024.10	1	26,024.10	1.01	0.3609
AC	3.101 × 10^5^	1	3.101 × 10^5^	12.04	0.0178
AD	5.537 × 10^5^	1	5.537 × 10^5^	21.51	0.0056
BC	2.849 × 10^5^	1	2.849 × 10^5^	11.07	0.0209
A^2^	3.466 × 10^6^	1	3.466 × 10^6^	134.63	<0.0001
B^2^	34,734.94	1	34,734.94	1.35	0.2979
C^2^	44,550.89	1	44,550.89	1.73	0.2455
A^2^B	7.940 × 10^5^	1	7.940 × 10^5^	30.84	0.0026
A^2^C	3.292 × 10^5^	1	3.292 × 10^5^	12.79	0.0159
AB^2^	7.288 × 10^5^	1	7.288 × 10^5^	28.30	0.0031
AC^2^	6.688 × 10^5^	1	6.688 × 10^5^	25.98	0.0038
B^2^C	1.389 × 10^5^	1	1.389 × 10^5^	5.40	0.0678
B^2^D	1.920 × 10^5^	1	1.920 × 10^5^	7.46	0.0412
C^2^D	5.521 × 10^5^	1	5.521 × 10^5^	21.44	0.0057
Residual	1.287 × 10^5^	5	25,746.65		
Cor Total	9.678 × 10^6^	23			
R^2^ = 0.9867, Adjacent R^2^ = 0.9388					

**Table 6:** Analysis of variance summary of hardness based on varying factors.

Source	Sum of Squares	df	Mean Square	F-Value	*p*-Value
Model	2048.64	15	136.58	14.03	0.0004
A—Filler Content	46.24	1	46.24	4.75	0.0609
B—Orientation angle	101.50	1	101.50	10.43	0.0121
C—Fill pattern	409.22	1	409.22	42.03	0.0002
D—Nozzle temperature	14.74	1	14.74	1.51	0.2535
AB	7.09	1	7.09	0.7281	0.4183
AC	207.36	1	207.36	21.30	0.0017
AD	116.37	1	116.37	11.95	0.0086
CD	66.15	1	66.15	6.79	0.0313
B^2^	166.84	1	166.84	17.14	0.0033
A^2^D	78.81	1	78.81	8.09	0.0216
AB^2^	53.95	1	53.95	5.54	0.0464
AD^2^	13.72	1	13.72	1.41	0.2693
B^2^C	16.14	1	16.14	1.66	0.2339
A^2^B^2^	328.75	1	328.75	33.77	0.0004
A^2^C^2^	17.50	1	17.50	1.80	0.2169
Residual	77.89	8	9.74		
Cor Total	2126.53	23			
R^2^ = 0.9634, Adjacent R^2^ = 0.8947					

**Table 7:** Summary table for the predictions.

Target Property	Significant Terms (ANOVA/RSM)	Adjusted R^2^ (ANOVA/RSM)	Best ML Model	RMSE	R^2^
Tensile strength	Filler content, orientation angle, fill pattern, nozzle temperature	0.9685	Gradient Boosting	0.4959 MPa	0.9779
Young’s modulus	Filler content, orientation angle, nozzle temperature	0.9867	Gradient Boosting	28.59MPa	0.9879
Hardness	Fill pattern, filler content, nozzle temperature	0.9634	Gradient Boosting	0.278 MPa	0.968

## Data Availability

The original contributions presented in this study are included in the article. Further inquiries can be directed to the corresponding author.

## Supplementary Materials

The following supporting information can be downloaded at: https://www.mdpi.com/article/10.3390/polym18040527/s1, Figure S1: Confusion matrix for the model accuracy measure for tensile strength using (a) multiple linear regression (MLR), (b) K-nearest neighbors (KNN), (c) support vector machine (SVM), (d) gradient boosting; Figure S2: SHAP Analysis and Feature Importance for tensile strength (a,b) multiple linear regression (MLR), (c,d) K-nearest neighbors (KNN), (e,f) support vector machine (SVM), (g,h) gradient boosting; Figure S3: Confusion matrix for the model accuracy measure for Young’s modulus using (a) multiple linear regression (MLR), (b) K-nearest neighbors (KNN), (c) support vector machine (SVM), (d) gradient boosting; Figure S4: SHAP Analysis and Feature Importance for Young’s modulus (a,b) multiple linear regression (MLR), (c,d) K-nearest neighbors (KNN), (e,f) support vector machine (SVM), (g,h) gradient boosting; Figure S5: Confusion matrix for the model accuracy measure for hardness using (a) multiple linear regression (MLR), (b) K-Nearest Neighbors (KNN) Regression, (c) Support Vector Machine (SVM), and (d) Gradient boosting; Figure S6: SHAP Analysis and Feature Importance for hardness (a,b) multiple linear regression (MLR), (c,d) K-nearest neighbors (KNN), (e,f) support vector machine (SVM), (g,h) gradient boosting.
