# Supplementary material for: Machine Learning-Enabled Optimization and Prediction of Mechanical Properties of 3D-Printed PLA Composites Filled with Rice Husk Biochar
Source: Polymers (Basel). 2026 Feb 21;18(4):527. doi: 10.3390/polym18040527 (PMC12944007; doi:10.3390/polym18040527)
Supplement: Supplementary file 1 [file polymers-18-00527-s001.zip › polymers-4126719-supplementary.pdf]

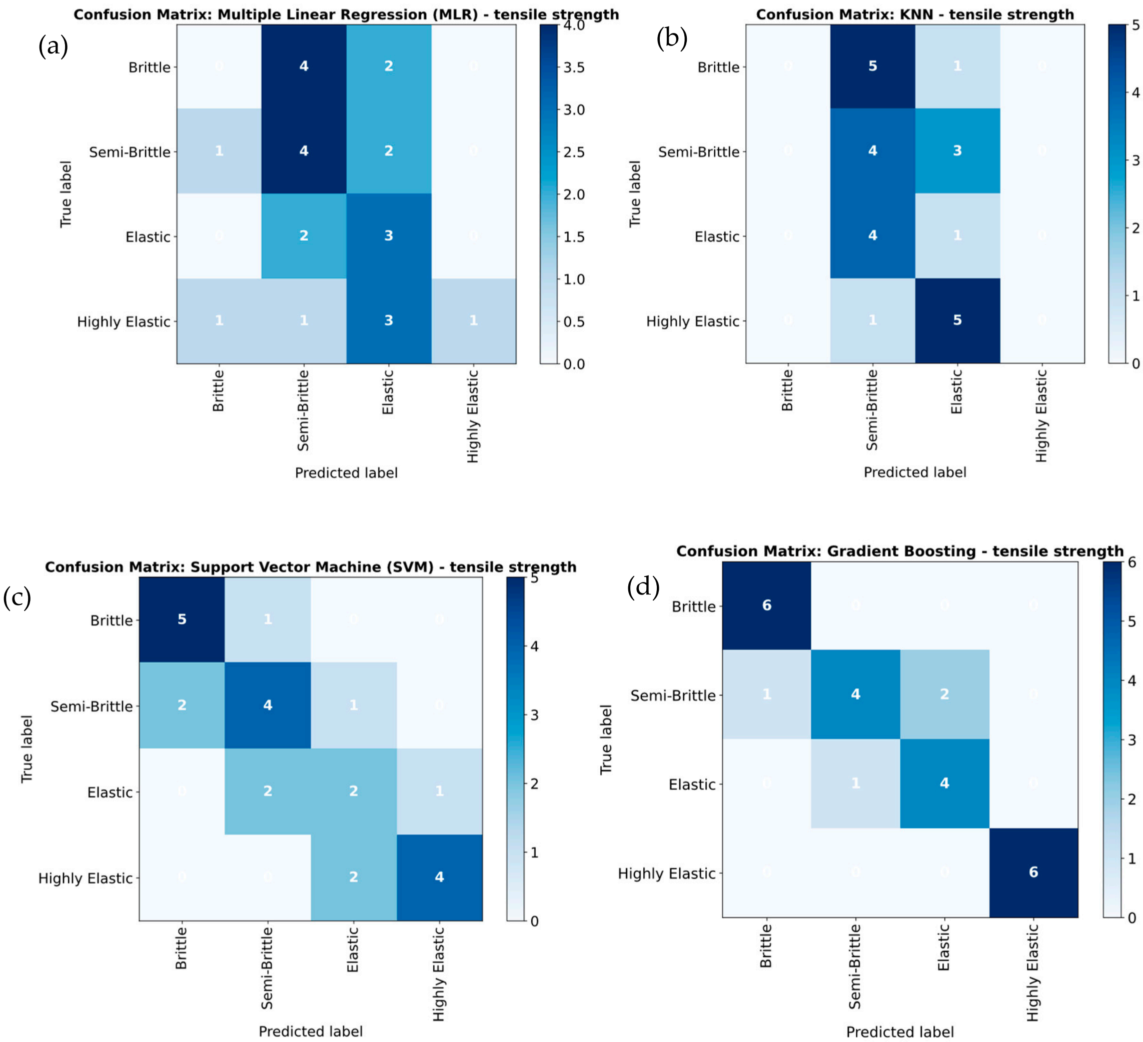

Figure S1 Confusion matrix for the model accuracy measure for tensile strength using a) multiple linear regression (MLR), b) K-nearest neighbors (KNN), c) support vector machine (SVM), d) gradient boosting

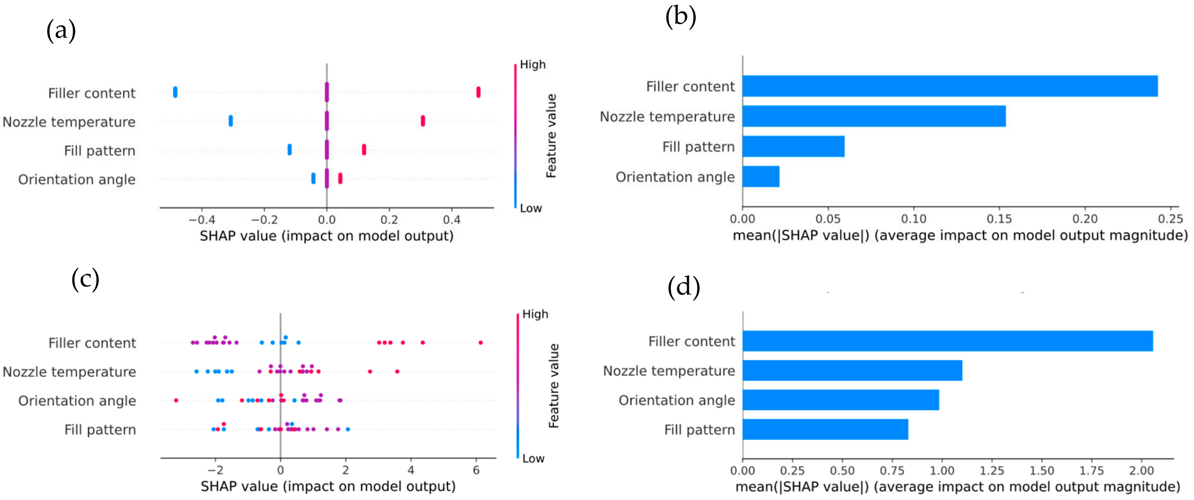

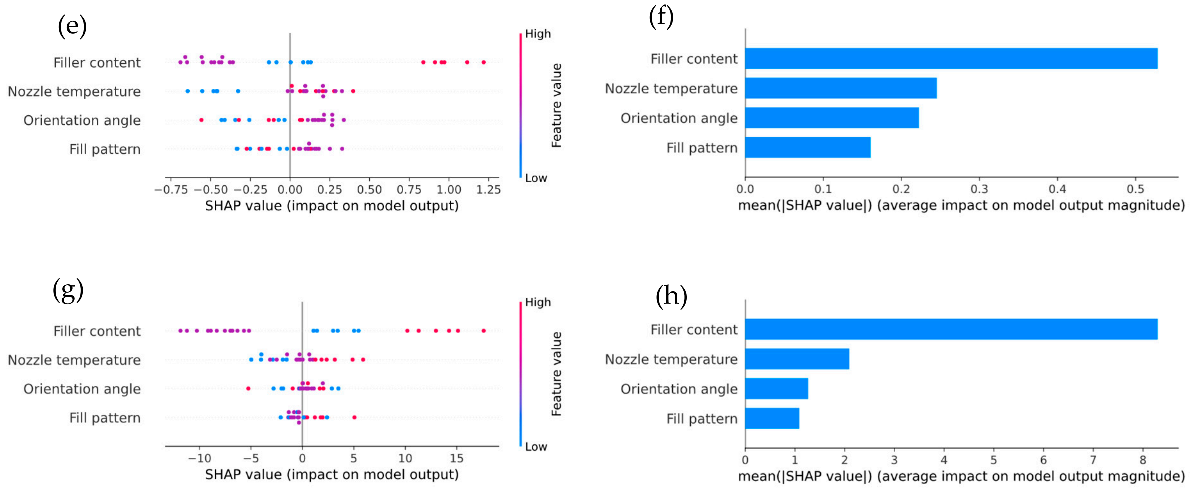

Figure S2. SHAP Analysis and Feature Importance for tensile strength a), b) multiple linear regression (MLR), c), d) K-nearest neighbors (KNN), e), f) support vector machine (SVM), g), h) gradient boosting

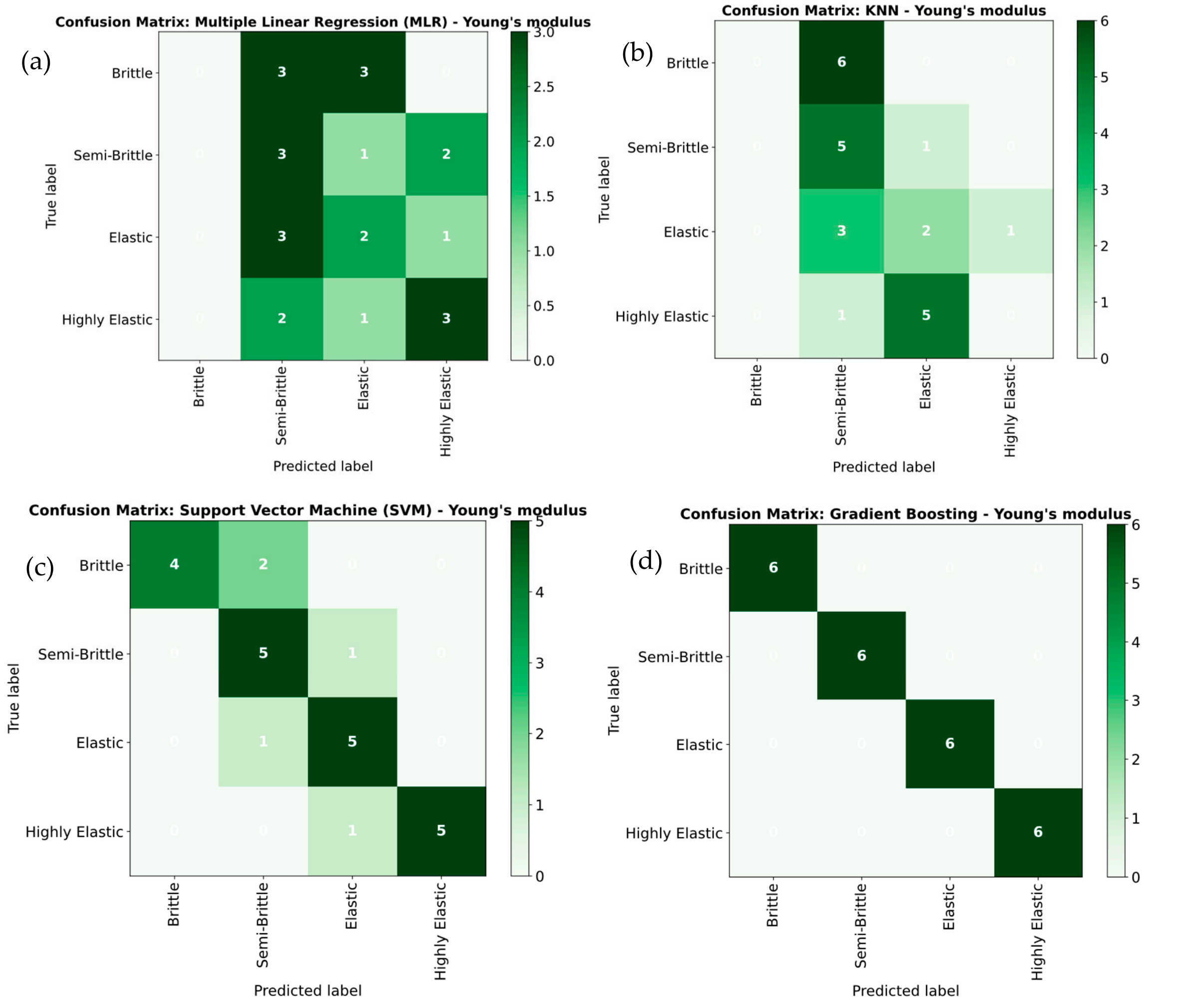

Figure S3. Confusion matrix for the model accuracy measure for Young's modulus using a) multiple linear regression (MLR), b) K-nearest neighbors (KNN), c) support vector machine (SVM), d) gradient boosting

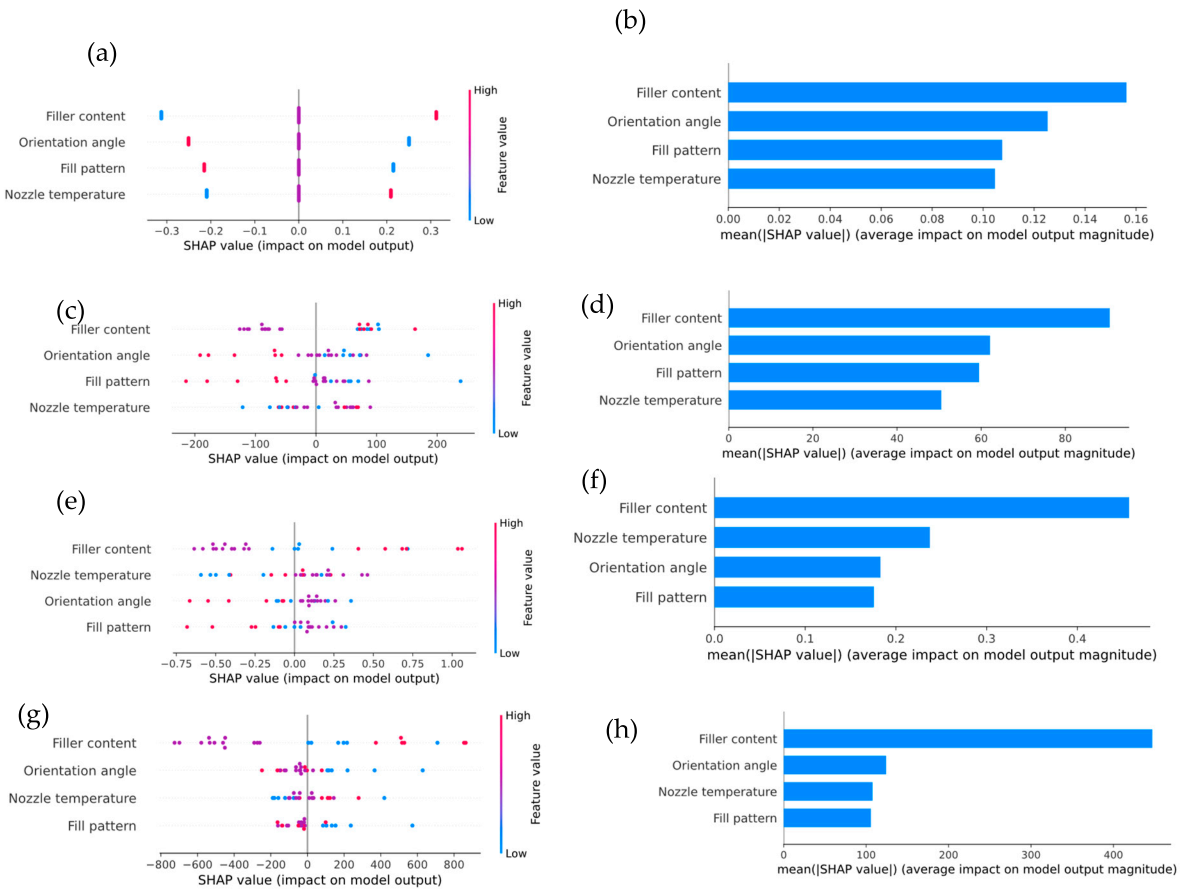

Figure S4. SHAP Analysis and Feature Importance for Young's modulus a), b)multiple linear regression (MLR), c), d) K-nearest neighbors (KNN), e), f) support vector machine (SVM), g), h) gradient boosting

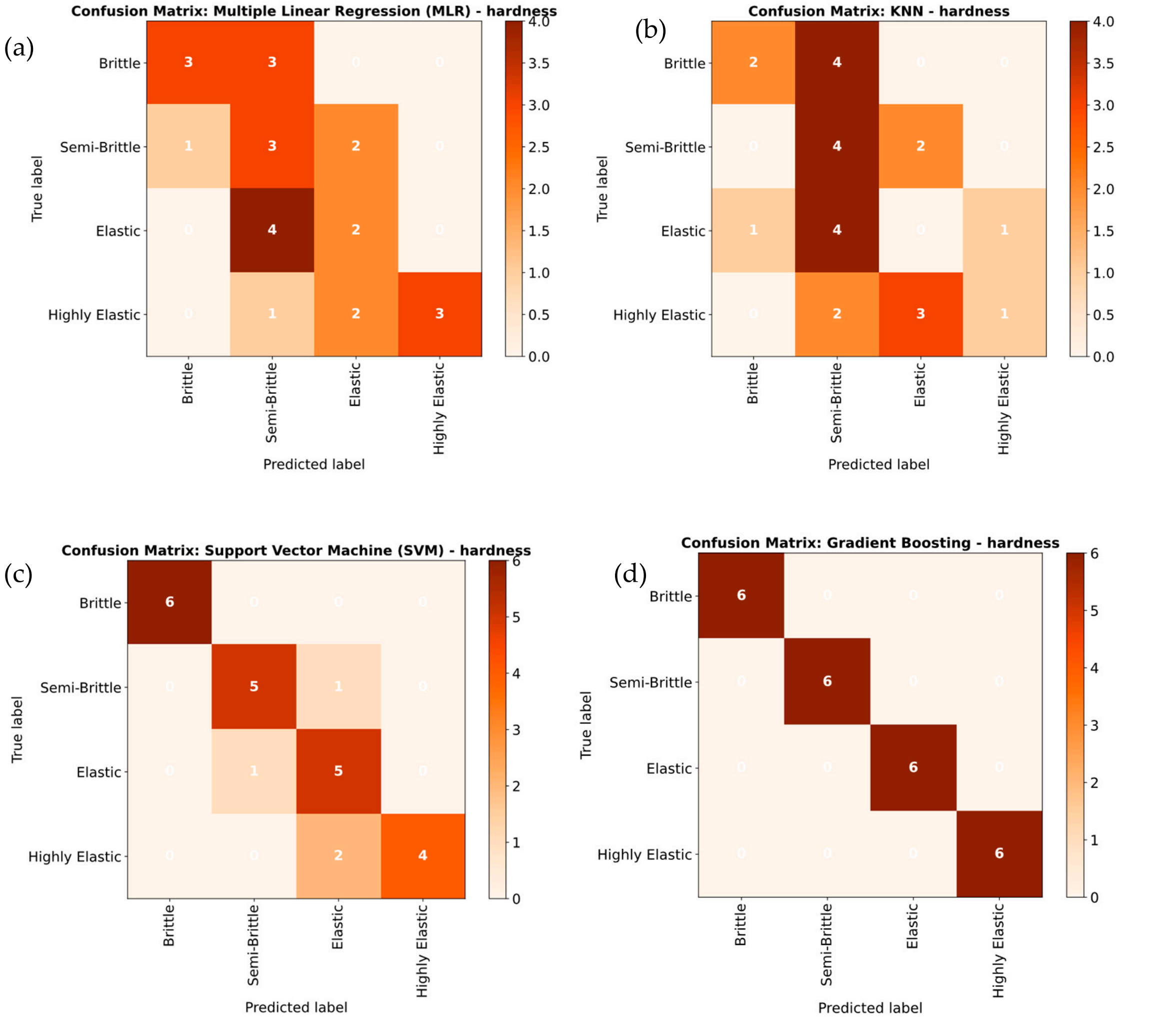

Figure S5. Confusion matrix for the model accuracy measure for hardness using (a) multiple linear regression (MLR), (b) K-Nearest Neighbors (KNN) Regression, (c) Support Vector Machine (SVM), and (d) Gradient boosting.

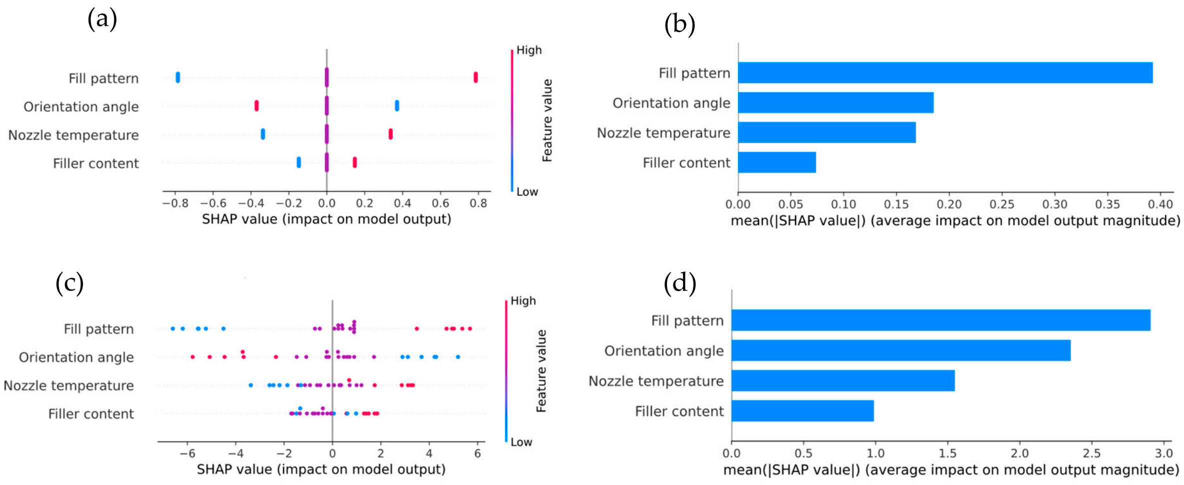

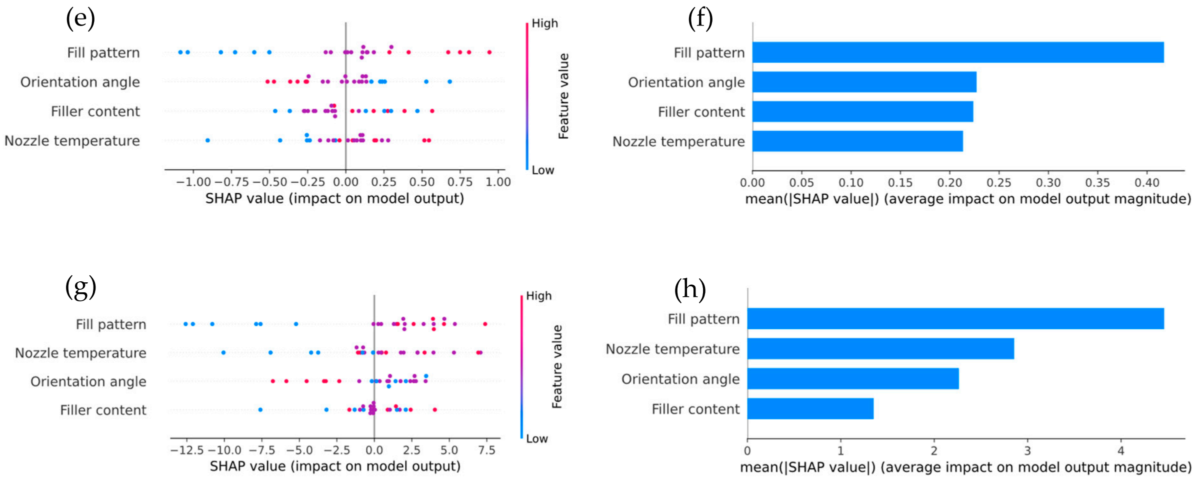

Figure S6. SHAP Analysis and Feature Importance for hardness a), b)multiple linear regression (MLR), c), d) K-nearest neighbors (KNN), e), f) support vector machine (SVM), g), h) gradient boosting
